# Ultraviolet photodissociation of methanethiol (CH_3_SH): revealing an S(^1^D) atom elimination channel

**DOI:** 10.1039/d5sc04716a

**Published:** 2025-08-18

**Authors:** Yucheng Wu, Shunyang Zhou, Zijie Luo, Shuaikang Yang, Zhenxing Li, Yongxin Dong, Wei Hua, Quan Shuai, Dongxu Dai, Michael N. R. Ashfold, Kaijun Yuan, Xueming Yang

**Affiliations:** a State Key Laboratory of Chemical Reaction Dynamics and Dalian Coherent Light Source, Dalian Institute of Chemical Physics, Chinese Academy of Sciences 457 Zhongshan Road Dalian 116023 China kjyuan@dicp.ac.cn; b University of Chinese Academy of Sciences Beijing 100049 China; c Marine Engineering College, Dalian Maritime University Liaoning 116026 China; d Institute of Advanced Light Source Facilities Shenzhen Guangdong 518100 China; e School of Chemistry, University of Bristol Bristol BS8 1TS UK mike.ashfold@bristol.ac.uk; f Hefei National Laboratory Hefei 230088 China; g Department of Chemistry and Center for Advanced Light Source Research, College of Science, Southern University of Science and Technology Shenzhen 518055 China

## Abstract

We report time-sliced velocity map imaging studies of the methyl (CH_3_) and electronically excited sulfur (S(^1^D)) fragments formed following the photoexcitation of jet-cooled CH_3_SH molecules in the 2^1^A′′ ← X̃ ^1^A′ absorption band (*i.e*. at wavelengths in the range 190 ≤ *λ* ≤ 210 nm). Analyses of images of CH_3_ fragments in their *v*_2_ = 0, 1 and 2 vibrational levels confirm the perpendicular parent transition dipole moment and prompt bond fission and show that the ground state SH(X) partners are formed with an inverted vibrational population distribution, peaking at *v* = 2 at the shortest excitation wavelengths investigated. Most of the photolysis photon energy above that required to break the C–S bond is partitioned into product translational energy. Primary S(^1^D) products are observed on excitation at *λ* ≤ 204 nm and their relative yield is deduced to increase quite steeply with decreasing wavelength, but quantum yield estimates are beyond the scope of the present work. Image analysis reveals that the CH_4_ partners are formed with a highly inverted vibrational population distribution, largely concentrated in the *ν*_4_ bending mode. A possible formation mechanism for the S(^1^D) + CH_4_ products is suggested, based on frustrated C–S bond extension on the initially populated 2^1^A′′ potential energy surface (PES) and re-collision between the embryonic CH_3_ and SH moieties in the extended region of conical intersection between the 2^1^A′′ and 1^1^A′′ PESs *en route* to the target products. Cutting edge electronic structure calculations along with complementary *ab initio* molecular dynamics studies should help validate or overturn this envisaged mechanism.

## Abstract 2

Time sliced velocity map imaging measurements reveal S atom elimination following ultraviolet photoexcitation of methanethiol. The CH_4_ partner fragments carry substantial vibrational excitation, concentrated in the *v*_4_ bending mode.

## Graphical abstract

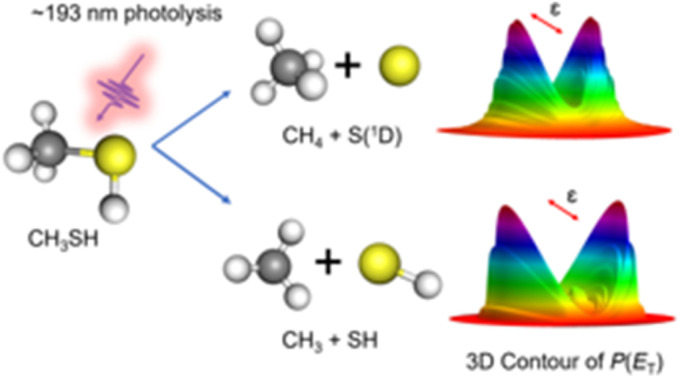

## 1. Introduction

Metabolization of dimethylsulfoniopropionate (DMSP) produced by phytoplankton and other marine organisms in seawater is recognised as the major biogenic source of dimethyl sulfide (DMS) on Earth. Once emitted into the atmosphere, DMS is rapidly oxidized to become an important precursor of sulfated aerosols and cloud condensation nuclei.^1^ Microbial action can also demethylate DMSP to methanethiol (CH_3_SH),^2^ but the roles of CH_3_SH in the oceans and the atmosphere are only now starting to be explored at levels of detail similar to that hitherto focused on DMS.^3–8^ The long wavelength end of the electronic absorption by CH_3_SH lies at ultraviolet (UV) wavelengths close to the short wavelength end of the solar spectrum that penetrates through the ozone layer, so CH_3_SH destruction in the troposphere is by reaction (notably oxidation by OH radicals^9^), not photochemistry.

More widely, sulfur is one of the more abundant elements in the universe. The S/H ratio in the solar photosphere is ∼1.3 × 10^−5^ (ref. 10), though the deduced quantity of S-containing species in dense clouds in the interstellar medium (ISM) is currently far lower than this quoted cosmic abundance.^11^ One plausible explanation for this apparent depletion is that much of the sulfur is incorporated and processed within dust grains and icy mantles. The relative abundance and mobility of hydrogen in an ice matrix suggests that most of the sulfur released by sputtering, thermal- or photo-desorption from such surfaces will be in the form of H_2_S,^12^ but similar mechanisms could give rise to CH_3_SH in the ISM.^13^ CH_3_SH was first detected tentatively,^14^ then definitively,^15^ towards the prolific high-mass star-forming region Sagittarius B2 close to the Galactic center, and has subsequently been observed in a range of locations, *e.g.* towards the organic hot-core G327.3-06,^16^ the cold core B1,^17^ the dense, warm part of a high-mass star-forming region in Orion,^18^ in the vicinity of the solar type protostar IRAS 16293-2422 (ref. 19) and the prestellar core L1544,^20^ in both the cold envelope and the hot gas around Cyg X-N12 (ref. 21) and in the hot gas surrounding G328.2551-0.5321.^22^ The tentative identification of DMS in the atmosphere surrounding exoplanet K2-18 (ref. 23) has fuelled debate regarding the possibility of life on planets other than Earth, but other recent observational^24^ and laboratory^25^ studies serve as a reminder that DMS may also arise *via* abiotic routes.

Photodissociation can be an important contributor to CH_3_SH destruction in many regions of the ISM. As noted above, the electronic absorption of CH_3_SH is concentrated in the UV spectral region,^26–29^ comprising a broad region of continuous absorption that peaks at *λ* ∼235 nm and extends to beyond 300 nm and more localised, structured Rydberg features at shorter wavelengths converging towards the first ionisation potential (IP = 9.2922 ± 0.0007 eV, corresponding to an excitation wavelength *λ* = 133.4 nm).^30^ Photoexcitation within the first absorption band promotes an electron from the highest occupied molecular orbital (HOMO, a non-bonding 3p orbital centred on the S atom) to an excited orbital with mixed Rydberg (4s)/valence (σ*) character.^31,32^ The resulting 1^1^A′′ excited state is dissociative with respect to both the H_3_CS–H and H_3_C–SH bond extension coordinates (henceforth *R*_S–H_ and *R*_C–S_, respectively), though the 1^1^A′′ potential energy surface (PES) displays a modest barrier at short C–S separations. Prompt S–H bond rupture is thus the dominant fate when exciting CH_3_SH at long wavelengths. The resulting CH_3_S(X̃) fragments are formed with modest vibrational excitation in the *ν*_3_ (C–S stretch) mode, but most of the photon energy in excess of that required to break the S–H bond (*D*_0_(H_3_CS–H) = 30 250 ± 100 cm^−1^, ref. 33) is partitioned into product translational motion along axes that are preferentially perpendicular to the transition dipole moment (and thus to the polarization vector **ε** of the photolysis laser radiation).^33,34^ The CH_3_(*v* = 0) fragment images recorded when exciting CH_3_SH in the range 220 ≤ *λ* ≤ 233 nm also display near limiting perpendicular recoil anisotropy and show that most of the partner SH(X) fragments are formed in their ground vibrational state.^35^ The relative yield of CH_3_ + SH products increases on tuning to shorter wavelengths,^36^ particularly when exciting the more localised 2^1^A′′ ← X̃^1^A′ band that dominates the absorption spectrum at wavelengths in the range 190 ≤ *λ* ≤ 215 nm, such that the two channels have similar quantum yields when photolyzing at *λ* = 193 nm.^37^

**Fig. 1:**
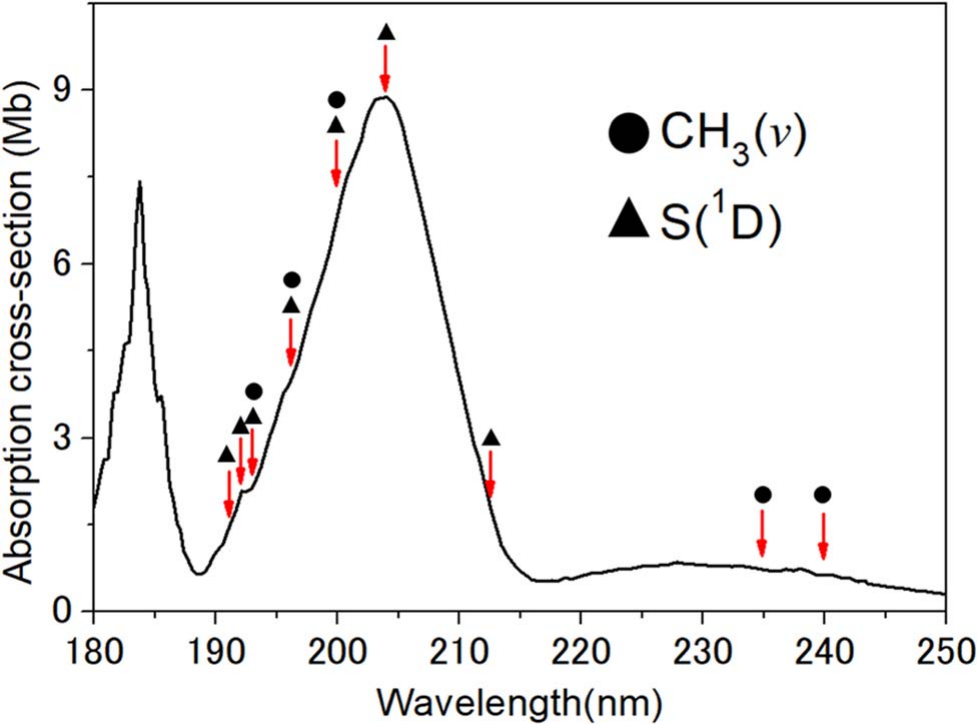
Room temperature absorption spectrum of CH_3_SH (after ref. 27 and 29), with the photolysis wavelengths investigated in the present work marked and labelled to show the species probed at each wavelength.

The present work involves a detailed study of photofragmentation pathways following excitation of CH_3_SH within the 2^1^A′′ ← X̃^1^A′ absorption band. The parent absorption spectrum at wavelengths around this transition is shown in Fig. 1, along with arrows showing the species probed at each wavelength investigated. This transition is attributed to a 4p/4s(a′) ← HOMO(a′′) electron promotion. The adiabatic 2^1^A′′ state is bound with respect to extending *R*_S–H_ or *R*_C–S_, but both dissociation coordinates can be accessed *via* efficient non-adiabatic coupling at a region of conical intersection (CI) between the 2^1^A′′ and 1^1^A′′ PESs in the near vertical Franck–Condon region.^31,32,38,39^ Prior studies at *λ* = 208 nm (ref. 33) and 193 nm (ref. 34) revealed substantial vibrational excitation in the CH_3_S(X̃) fragments from the former channel, most notably an obvious progression in the *ν*_3_(C–S) stretch mode. This points to some significant coupling between the C–S and S–H stretch motions during the dissociation process and the likely inadequacy of any one-dimensional representation of what is an inherently multi-dimensional problem.^38,39^ CH_3_ fragments from the latter channel have been detected, generally with vibrational state specificity, following excitation at several wavelengths in the range 202 ≤ *λ* ≤ 210 nm.^35,40–42^ The SH(X) partners formed at these shorter excitation wavelengths populate a range of vibrational (*v*) states, with distributions that peak at *v* > 0. The products from both channels again display preferential perpendicular recoil anisotropy, implying that non-adiabatic coupling *via* the 2^1^A′′/1^1^A′′ CI is efficient and that dissociation occurs on a timescale comparable to, or faster than, the rotational period of the jet-cooled parent CH_3_SH molecules.

Dissociation channels (1) and (2) are just two of many thermochemically allowed decay channels available to CH_3_SH molecules when excited at *λ* ∼200 nm. Focusing on spin-allowed fragmentation processes yielded two products,

1 CH_3_SH + hν → CH_3_ + SH *E*_th_ = 25340 ± 40 cm^−1^

2 → CH_3_S + H *E*_th_ = 30250 ± 100 cm^−1^,

could be supplemented by one or more of the following fragmentations

3 CH_3_SH + hν → H_2_CS + H_2_ *E*_th_ = 11000 ± 200 cm^−1^

4 → S(^1^D) + CH_4_ *E*_th_ = 27690 ± 40 cm^−1^

5 → CH_2_(ã^1^A_1_) + H_2_S *E*_th_ = 35260 ± 40 cm^−1^,

and dissociations yielding products in higher excited states, *e.g*.

6 CH_3_SH + hν → H_2_CS(Ã) + H_2_ *E*_th_ = 27400 ± 200 cm^−1^

7 → S(^1^S) + CH_4_*E*_th_ = 40630 ± 40 cm^−1^,

though we recognise that the energetic onsets for many of these later eliminations may be higher than these quoted thermochemical threshold (*E*_th_) values as the process is likely to involve passage over an energy barrier. Inclusion of spin forbidden dissociations yielding, *e.g*., S(^3^P) atoms or triplet state CH_2_ radicals or excited triplet state H_2_CS molecules (processes not currently included in this list) opens the range of possible dissociation processes yet further, and the onset of the lowest energy three-body dissociation channel (8) is only just above the energy of a *λ* = 200 nm photon.

8 CH_3_SH + hν → CH_3_ + S(^3^P) + H *E*_th_ = 53730 ± 40 cm^−1^.

The *D*_0_(H_3_CS–H) value quoted for dissociation (2) is from ref. 33. All other threshold energies quoted here are derived using Δ_f_*H*°(0 K) values and uncertainties from ref. 43 and atomic term values from ref. 44, while the Ã–X̃ term value for thioformaldehyde used in determining *E*_th_(6) is from Jacox.^45^

H_2_CS formation was predicted in early photolysis studies of CH_3_SH at *λ* = 185 nm (ref. 46) and identified in later time-of-flight mass spectrometry measurements following CH_3_SH photolysis at the ArF laser wavelength (193.3 nm).^47^ S(^3^P) and S(^1^D) atoms have been detected following 193.3 nm excitation of CH_3_SH but, from measurements of the signal dependence upon photolysis laser intensity, these were deduced to arise from secondary photolysis of primary CH_3_S and/or SH photoproducts.^48^ The present work extends high resolution imaging measurements of the CH_3_(*v*) products down to the short wavelength end of the 2^1^A′′ ← X̃^1^A′ absorption band, *i.e. λ* ≥ 191 nm (Fig. 1), thereby enabling new insights into the dissociation process (1), and provides first and definitive evidence for the operation of the rival dissociation channel (4) yielding S(^1^D) + CH_4_ products.

## 2. Experimental

The experiments employed a time-sliced velocity map ion imaging (TS-VMI) detection apparatus, details of which have been reported previously,^49–54^ along with UV photolysis laser pulses in the range 190 ≤ *λ* ≤ 240 nm and both UV and vacuum UV (VUV) probe laser pulses to detect, respectively, CH_3_(*v*) and S(^1^D) photofragments. The CH_3_SH sample was introduced into the source chamber as a pulsed supersonic beam (10% CH_3_SH in Ar, Kylingas (99.5% purity)), where it was skimmed prior to entering (through a 2 mm hole in the first electrode) and propagating along the centre axis of the 23-plate ion optics assembly (IOA) within the spectrometer. The photolysis and probe laser beams intercepted the pulsed molecular beam at right angles, between the second and third plates of the IOA.

The requisite UV photolysis wavelengths were generated by frequency doubling or sum-frequency mixing using a table-top laser system. Light at *λ* ≥ 204 nm (∼1 mJ per pulse, pulse duration ∼10 ns) was produced by frequency doubling the output of a dye laser (Sirah, PESC-G-24) pumped by the third harmonic (355 nm) output of a Nd:YAG laser (Continuum PL-9030). As such, the photolysis photon wavenumber in all cases was defined to sub-cm^−1^ precision; the wavelengths used were chosen to be a whole number of nm. Light in the wavelength range 191 ≤ *λ* ≤ 199 nm (∼0.3 mJ per pulse, pulse duration ∼10 ns) was generated as the sum (*i.e. ω*_1_ + *ω*_2_) frequency output using a BBO crystal, with *ω*_1_ set at the frequency corresponding to *λ*_1_ = 266 nm (produced by frequency doubling the output of a 355 nm (the third harmonic output of a Continuum PL-9030 Nd:YAG laser) pumped dye laser (Sirah, PESC-G-24 operating at *λ* ∼532 nm). The requisite *ω*_2_ frequencies were produced using half of the second harmonic (532 nm) output from the same Continuum Nd:YAG laser to pump another dye laser (Sirah, PESC-G-24) generating photons with *λ*_2_ wavelengths in the range 677–790 nm (∼8–10 mJ per pulse, duration of ∼10 ns).

S(^1^D) photoproducts were probed by Doppler scanning back and forth across the one photon absorption at *λ* = 130.091 nm, which populates the autoionizing 3p^3^(^2^D°)5s; 
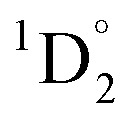
 level of atomic sulfur. As previously reported,^50,55,56^ these VUV photons were generated by four wave difference (*i.e*. 2*ω*_3_ − *ω*_4_) frequency mixing of the frequency doubled output from one dye laser (at *λ*_3_ = 212.556 nm) with the fundamental output of a second dye laser (at *λ*_4_ = 580.654 nm) in a Kr/Ar gas mixture. The contrast between the structured and underlying continuum two-colour contributions to the S(^1^D) images was boosted by deliberately shifting the focus of the photolysis laser radiation 8 cm away from the interaction region, by translating the position of the 50 cm focal length lens used to focus this radiation. CH_3_(X̃, *v*) photoproducts were probed by two photon resonance enhanced multiphoton ionization (2 + 1 REMPI) using documented excitation wavelengths in the range 325 ≤ *λ* ≤ 333.5 nm to sample population in the *v* = 0, *v*_2_ = 1, *v*_2_ = 2 and *v*_1_ = 1 levels,^57,58^ as described elsewhere.^59^ The requisite UV pulses (∼2 mJ per pulse, duration of ∼10 ns) were produced by frequency doubling the output of another (Sirah, PESC-G-24) dye laser operating at *λ* ∼650–667 nm, which was pumped by the other half of the 532 nm output from the above Nd-YAG laser. The polarization vectors of the photolysis (**ε**_**phot**_) and probe (**ε**_**probe**_) laser radiation were both parallel to the front face of the detector, and the crossing angle between the photolysis and REMPI probe laser beam paths in the interaction region was ∼7°, where the time delay between the respective pulses was in the range 10–20 ns.

The resulting S^+^/CH_3_^+^ ions were accelerated through the rest of the IOA and detected with a dual microchannel plate assembly coupled with a P43 phosphor screen at the end of the 740 mm ion flight tube. The detector was time gated (15–20 ns) to select ions with *m*/*z* 32 (S^+^) or 15 (CH_3_^+^) and to confirm that the signal was from the intended two-colour UV photolysis – REMPI/VUV probe scheme, and three images were recorded for each set of experimental conditions with: (i) both the photolysis and probe beams present in the interaction region; (ii) the photolysis beam present but the probe beam blocked, and (iii) the photolysis beam blocked and the probe beam present. For all two-colour images displayed in this article, the one-colour photoinduced background images recorded under conditions (ii) and (iii) have already been subtracted from the image recorded under condition (i).

## 3. Results

### 3.1 CH_3_ fragment imaging

**Fig. 2:**
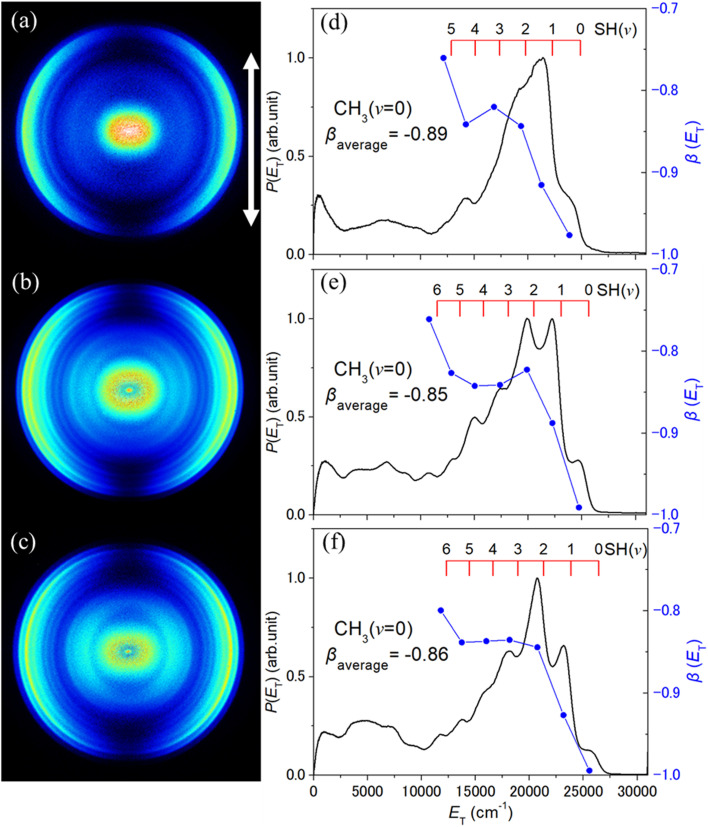
TS-VM images of CH_3_(*v* = 0) fragments formed by photolysis of jet-cooled CH_3_SH molecules at *λ* = (a) 199, (b) 196 and (c) 193 nm, with **ε_phot_** aligned vertically in the plane of the image as indicated by the double headed arrow in (a). The corresponding *P*(*E*_T_) (black) and *β*(*E*_T_) (blue) distributions derived from each image are displayed in panels (d)–(f), with the relevant scales shown on, respectively, the left- and right-hand *y*-axes, along with the *β*_average_ value determined over the indicated *E*_T_ range. The red combs above the respective *P*(*E*_T_) spectra indicate the maximum *E*_T_ value for the specified co-fragment formed with the probed CH_3_(*v* = 0) species.

Fig. 2 shows TS-VM images of the CH_3_(*v* = 0) fragments obtained following photolysis of the jet-cooled CH_3_SH sample at *λ* = 199, 196 and 193 nm, at the high energy end of the 2^1^A′′ ← X̃^1^A′ absorption band. Corresponding images of CH_3_ products formed in the *v*_2_ = 1 and *v*_2_ = 2 levels (where *ν*_2_ is the out-of-plane umbrella bending mode) are shown in Fig. S1 and S2 in the SI. In all cases, **ε**_**phot**_ is in the plane of the image, as shown by the double headed arrow included in panel (a) of each of these figures. Analysis of such images allows determination of (i) the total kinetic energy release, *P*(*E*_T_), distributions, given the provisos of two body dissociation, momentum conservation and the partner fragment being SH, and (ii) the (*E*_T_-dependent) recoil anisotropy. The latter is defined in terms of the anisotropy parameter, *β*, which is obtained by fitting the measured intensities to the expression *I*(*θ*) ∝ [1 + *β*(*P*_2_(cos *θ*))], where *θ* defines the angle of the recoil velocity vector relative to **ε**_**phot**_ and *P*_2_(cos *θ*) is the second Legendre polynomial. *β* takes limiting values of +2 and −1 in the case of axial recoil following one photon excitation *via* a transition dipole moment that lies, respectively, parallel and perpendicular to the breaking bond. The *P*(*E*_T_) and *β*(*E*_T_) plots (black and blue, respectively) are shown to the right of the corresponding images in Fig. 2, S1 and S2.

Each image shows a structured, anisotropic ring at a large radius and a secondary feature concentrated at the image centre. The former arises from the target process (1). The latter feature has been observed previously following excitation at other wavelengths in this region,^35,41,42^ and is logically attributed to CH_3_ fragments arising *via* unwanted probe-laser-induced photolysis of CH_3_S(X̃) fragments formed *via* the rival primary fragmentation channel (2).

We consider this latter signal first. The thermochemical threshold energy for process (9)

9 CH_3_S + hν → CH_3_ + S(^3^P) *E*_th_ = 24340 ± 100 cm^−1^

can be derived from the documented *D*_0_(H_3_CS–H) value^33^ and the relevant Δ_f_*H*°(0 K) values given in ref. 43. The wavelength of the probe photons used for CH_3_(*v* = 0) detection (*λ* = 333.46 nm, corresponding to a wavenumber of 29 988.6 cm^−1^) falls within the CH_3_S(Ã–X̃) absorption band.^60–62^ The resulting CH_3_S(Ã) fragments predissociate to CH_3_ + S(^3^P) products (process (9)), most of which display preferential perpendicular recoil anisotropy.^63,64^ CH_3_(*v* = 0) fragments arising *via* one probe laser photon induced photolysis of primary CH_3_S(X̃, *v* = 0) fragments should thus appear with *E*_T_ ∼5600 cm^−1^. Several factors serve to blur such an analysis for the present experiments. The primary CH_3_S(X̃) fragments from *λ* ∼200 nm photolysis of CH_3_SH are formed with a range of kinetic energies, though momentum conservation ensures that most of any *E*_T_ released in dissociation process (2) is carried by the light H atom partner. More importantly, the CH_3_S(X̃) fragments are distributed over a range of vibrational states, some with internal energies (*E*_int_) approaching the limit allowed by energy conservation.^33,34^ The relative absorption cross-sections of CH_3_S(X̃) fragments in different *v* levels at the probe wavelength of interest are not known, nor are many details of the dissociation dynamics of different CH_3_S(Ã, *v*) fragments. Conceivably, however, the probe-laser induced photodissociation of primary CH_3_S(X̃) fragments in high *E*_int_ states could yield CH_3_ + S(^3^P) products with *E*_T_ ∼15 000 cm^−1^ or more, which would overlap with features attributable to CH_3_ + SH(X, higher *v*) products (primary process (1)) in the *P*(*E*_T_) spectra shown in Fig. 2, S1 and S2. The CH_3_ fragments attributed to probe-laser induced photolysis of the ensemble of primary CH_3_S(X̃) products show some preferential perpendicular recoil anisotropy (Fig. S1).

We now focus on the feature at a larger radius in these CH_3_(*v*) images. As shown by the combs superposed above the *P*(*E*_T_) distributions derived from the CH_3_(*v* = 0), CH_3_(*v*_2_ = 1) and CH_3_(*v*_2_ = 2) images shown in Fig. 2, S1 and S2, the partner SH(X) fragments are formed in levels with *v* up to ∼6. For any set of CH_3_(*v*; *λ*) images, the onset at high *E*_T_ associated with the formation of SH(*v* = 0) co-fragments shows the expected shift to higher *E*_T_ as the photolysis wavelength is reduced. More strikingly, the most populated level in the SH(*v*) population distribution, *P*(*v*), clearly increases from *v* = 1 to *v* = 2 as the photolysis wavelength is decreased from 199 nm to 193 nm, but the various *P*(*v*) distributions appear to be relatively insensitive to the probed CH_3_(*v*) level. No signal attributable to CH_3_(*v*_1_ = 1) fragments was observed when excited at the probe wavelength recommended^58^ for their detection. The SH product vibrational energy distributions can be put on a more quantitative footing by fitting the higher-*E*_T_ part of the *P*(*E*_T_) distributions using a set of suitably positioned Gaussian functions to represent the different SH(*v*) levels. An illustrative decomposition of the data obtained at *λ* = 196 nm is shown in Fig. S3. Of note, the signal attributable to the secondary photolysis of primary CH_3_S fragments is likely to extend to *E*_T_ values attributed to primary CH_3_ + SH(X, *v* = 5 and, particularly, 6) products, so the present analysis almost certainly overestimates the relative yields of these SH(X, high *v*) levels. The overlapping contributions from these two CH_3_ radical sources may be responsible for the apparent reduction in the recoil anisotropy of the CH_3_ + SH(X, higher *v*) products.

**Fig. 3:**
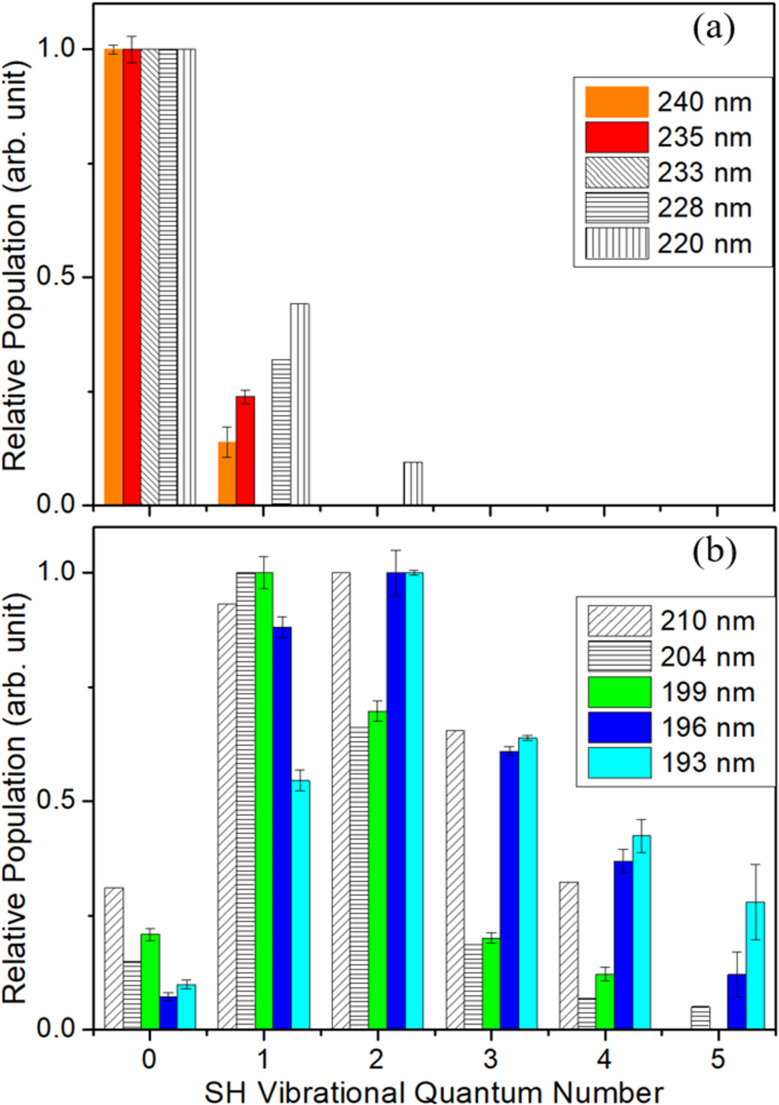
SH *P*(*v*) distributions from photolysis of a jet-cooled CH_3_SH sample deduced from analysing CH_3_(*v* = 0) images measured at *λ* = (a) 240 and 235 nm (present work) and 233, 228 and 220 nm (from ref. 35) and (b) 210 nm (ref. 42), 204 nm (ref. 41), 199, 196 and 193 nm (present work), in each case normalized such that the most populated level is plotted with unit intensity. The error bars represent the standard error in the simulation, but do not allow for the likely overestimation of the relative population in the highest *v* levels (see Section 3.1).

Fig. 3(b) shows the SH *P*(*v*) distributions deduced from analysing the CH_3_(*v* = 0) images obtained at *λ* = 199, 196 and 193 nm. These data are supplemented by the corresponding *P*(*v*) distributions reported previously following photolysis at *λ* = 204 nm (ref. 41) and 210 nm.^42^ SH *P*(*v*) distributions determined when excited at longer wavelengths (in the 1^1^A′′ ← X̃^1^A′ absorption continuum) are shown in Fig. 3(a). The *λ* = 235 and 240 nm data are derived from *P*(*E*_T_) spectra recorded as part of the present study (shown in Fig. S4 in the SI), while the SH *P*(*v*) distributions from CH_3_SH photolysis at *λ* = 220, 228 and 233 nm are from ref. 35. These long wavelength data are peripheral to the primary focus of the present paper but are included for completeness. All serve to reinforce previous conclusions that (i) the SH(X) fragments formed by photolysis within the 1^1^A′′←X̃^1^A′ continuum carry modest vibrational (and modest rotational) excitation, whereas those formed by photolysis in the 2^1^A′′ ← X̃^1^A′ band show inverted *P*(*v*) distributions, (ii) the energy disposal in the SH(X) fragments formed at any given wavelength is relatively insensitive to any vibrational excitation in the probed CH_3_(*v*_2_) fragment, and (iii) most of the photon energy in excess of that required to break the C–S bond is partitioned into product translational motion.

### 3.2 S(^1^D) fragment imaging

**Fig. 4:**
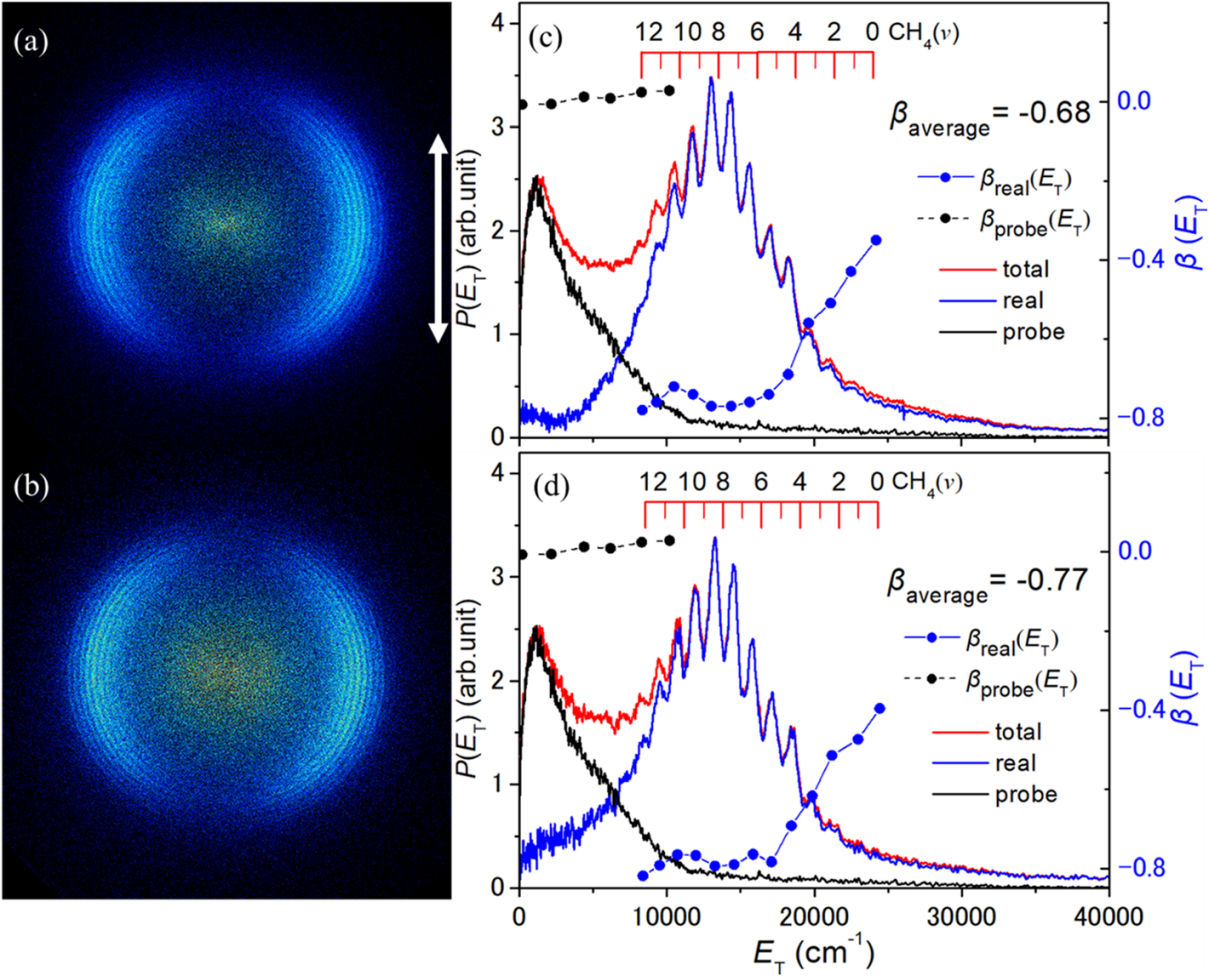
TS-VM images of the S(^1^D) fragments formed by photolysis of jet-cooled CH_3_SH molecules at *λ* = (a) 192 and (b) 191 nm, with **ε_phot_** aligned vertically in the plane of the image as indicated by the double headed arrow in (a). Panels (c) and (d) show the *P*(*E*_T_) distributions derived from this two-colour image (red trace), from the 130.091 nm probe-laser only image (black trace) and the ‘real’ pump–probe two-colour *P*(*E*_T_) distribution obtained from the difference (blue trace), referenced to the left-hand *y*-axis scale. The black and blue dots show the *β*(*E*_T_) distributions derived from the corresponding traces, referenced to the right-hand *y*-axis scale. The *β*_average_ value for the signal from photolysis laser induced dissociation determined over the indicated *E*_T_ range is also included in the inset. The red combs in panels (c) and (d) show the maximum *E*_T_ values for different S(^1^D) + CH_4_(*v*_4_) product channels.

**Fig. 5:**
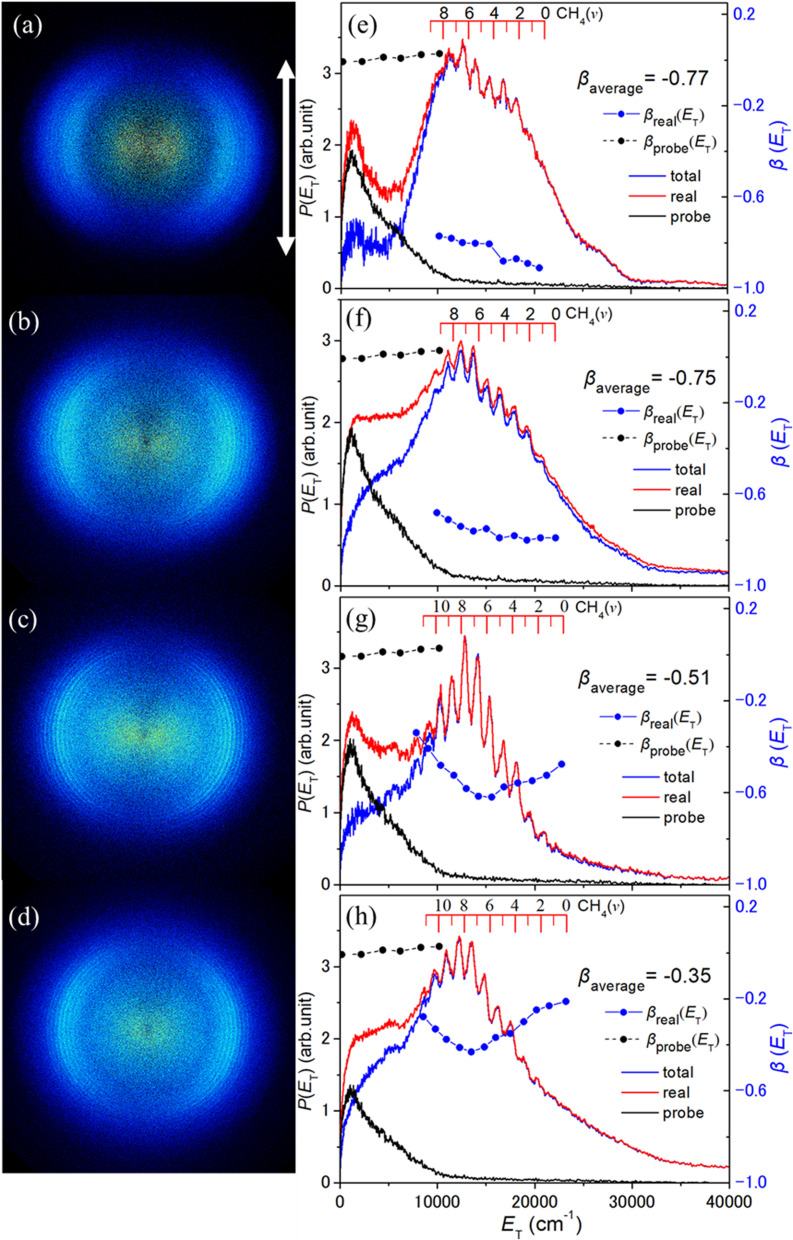
TS-VM images of the S(^1^D) fragments formed by photolysis of jet-cooled CH_3_SH molecules at *λ* = (a) 204, (b) 199, (c) 196 and (d) 193 nm, with **ε_phot_** aligned vertically in the plane of the image as indicated by the double headed arrow in (a). Panels (e)–(h) show the *P*(*E*_T_) distributions derived from each two-colour image (red trace), from the 130.091 nm probe-laser only image (black trace) and the ‘real’ pump–probe two-colour *P*(*E*_T_) distribution obtained from the difference (blue trace), referenced to the left-hand *y*-axis scale. The black and blue dots show the *β*(*E*_T_) distributions derived from the corresponding traces, referenced to the right-hand *y*-axis scale. The *β*_average_ value for the signal from photolysis laser induced dissociation determined over the indicated *E*_T_ range is also included in the inset. The red combs in panels (c) and (d) show the maximum *E*_T_ values for different S(^1^D) + CH_4_(*v*_4_) product channels.

Fig. 4(a) and (b) show TS-VM images of the S(^1^D) fragments formed when photolyzing jet-cooled CH_3_SH molecules at *λ* = 192 and 191 nm. As noted in the Experimental section, the focus of the photolysis laser radiation was translated 8 cm from the interaction region to maximise the contrast between the structured signal of most interest from the underlying photolysis-laser-induced background signal. Even a cursory examination of these images shows that the S(^1^D) images recoil along axes perpendicular to **ε_phot_**. The left-hand panels in Fig. 5 show the corresponding S(^1^D) fragment images measured under the same experimental conditions when exciting at *λ* = (a) 204, (b) 199, (c) 196 and (d) 193 nm. No fine structure was evident in the corresponding image measured at *λ* = 212 nm, as shown in Fig. S5. The panel to the right of each image shows the corresponding *P*(*E*_T_) distributions derived from (i) the two-colour image measured with both photolysis and probe lasers present (red trace), (ii) the one-colour probe laser only image (black trace), which peaks at low *E*_T_, and (iii) the difference (*i.e*. red minus black) distribution attributable to the real two-colour signal (blue trace). The *β*(*E*_T_) distribution derived from this real two-colour signal is also shown in each right-hand panel (blue points, joined by a dashed line) and the *β*(*E*_T_) distribution for the one colour probe only signal is also included in Fig. 4, 5 and S5 (black dots).

The two-colour images measured at *λ* ≤ 204 nm contain (at least) three components. The structure attributable to the S(^1^D) + CH_4_ products formed *via* channel (4) is the feature of greatest novelty but we choose to interpret the overlapping unstructured contributions first. The probe-only signal (black traces in Fig. 4, 5 and S5) is attributable to the probe (*λ* = 130.091 nm) laser induced three body dissociation

10 CH_3_SH + hν → CH_3_ + S(^1^D) + H *E*_th_ = 62970 ± 40 cm^−1^,

products from which, by energy conservation arguments, they could appear with *E*_T_ ≤ 13 900 cm^−1^. We note that the *E*_T_ values in the *P*(*E*_T_) traces displayed in Fig. 4, 5 and S5 are derived assuming two body dissociation and that the partner to the imaged S atom has *m* = 16, and so is not strictly accurate for products arising *via* process (10). But the black trace in each plot sensibly declines to the baseline by this predicted upper limit *E*_T_ value and peaks at much lower translational energies, implying significant internal excitation in the CH_3_ products formed at this VUV wavelength.

The second unstructured contribution lies under the structured features in the real two-colour signal (the blue traces in the displayed *P*(*E*_T_) spectra) and extends to *E*_T_ values much higher than that accessible *via* one photolysis photon induced formation of S(^1^D) + CH_4_ products. This contribution dominates the spectra recorded at longer wavelengths and is likely responsible for all the two-colour signal in the image measured at *λ* = 212 nm (Fig. S5). As Fig. S5 shows, the high *E*_T_ limit of this signal is sensibly consistent with that expected from resonance enhanced two-photolysis-photon induced dissociation of CH_3_SH *via* channel (10), *i.e*. *E*_T_ ≤ 31 370 cm^−1^ at *λ* = 212 nm. Inspection of the corresponding two-colour *P*(*E*_T_) spectra (blue traces) obtained at shorter wavelengths shows this high energy tail stretching out to progressively high *E*_T_ values consistent with the thermochemical prediction that two-photolysis-photon induced dissociation could yield products with *E*_T_ ≤ 35 070 cm^−1^ at *λ* = 204 nm and *E*_T_ ≤ 37 530 cm^−1^ at *λ* = 199 nm (Fig. 5). But inspection of Fig. 4 also suggests that this two-photon contribution is comparatively minor in the S(^1^D) product images measured at *λ* = 191 or 192 nm, *i.e.* that process (4) – which we will now show to be responsible for the structure in these *P*(*E*_T_) spectra – contributes only weakly at *λ* = 204 nm but its relative importance increases steeply on tuning to shorter excitation wavelengths within the 2^1^A′′ ← X̃^1^A′ absorption band.

From here on, we focus on the structured signal attributed to S(^1^D) + CH_4_ product formation. CH_4_ has four normal modes of vibration, which can be labelled by irreducible representations of the *T*_d_ point group, according to the symmetry of the associated normal coordinates. These are the symmetric (*ν*_1_) and asymmetric (*ν*_3_) stretching modes, with respective degeneracies of 1 and 3, and the *ν*_2_ and *ν*_4_ bending modes, with respective degeneracies of 2 and 3. The wavenumbers of the two stretch fundamentals are similar. The wavenumbers of the *ν*_2_ and *ν*_4_ fundamentals are also similar, and both are about half that of *ν*_1_ and *ν*_3_. This leads to a well-defined polyad structure, with each polyad *P*_*n*_ defined by the integer *n*, where

11 *n* = 2(*v*_1_ + *v*_3_) + *v*_2_ + *v*_4_.

The *v*_*i*_ in eqn (11) are the vibrational quantum numbers, and each (*v*_1_, *v*_2_, *v*_3_, *v*_4_) set defines a vibrational level, most of which – because of the degeneracies of the *ν*_2_, *ν*_3_ and *ν*_4_ modes – split into multiple sub-levels. The levels within each polyad span a range of energies, the spread of which increases quite rapidly with *n*.^65,66^ For future reference, we also note that the lowest energy levels within any *P*_*n*_ polyad are those associated with the *nv*_4_ levels.^65,66^

**Fig. 6:**
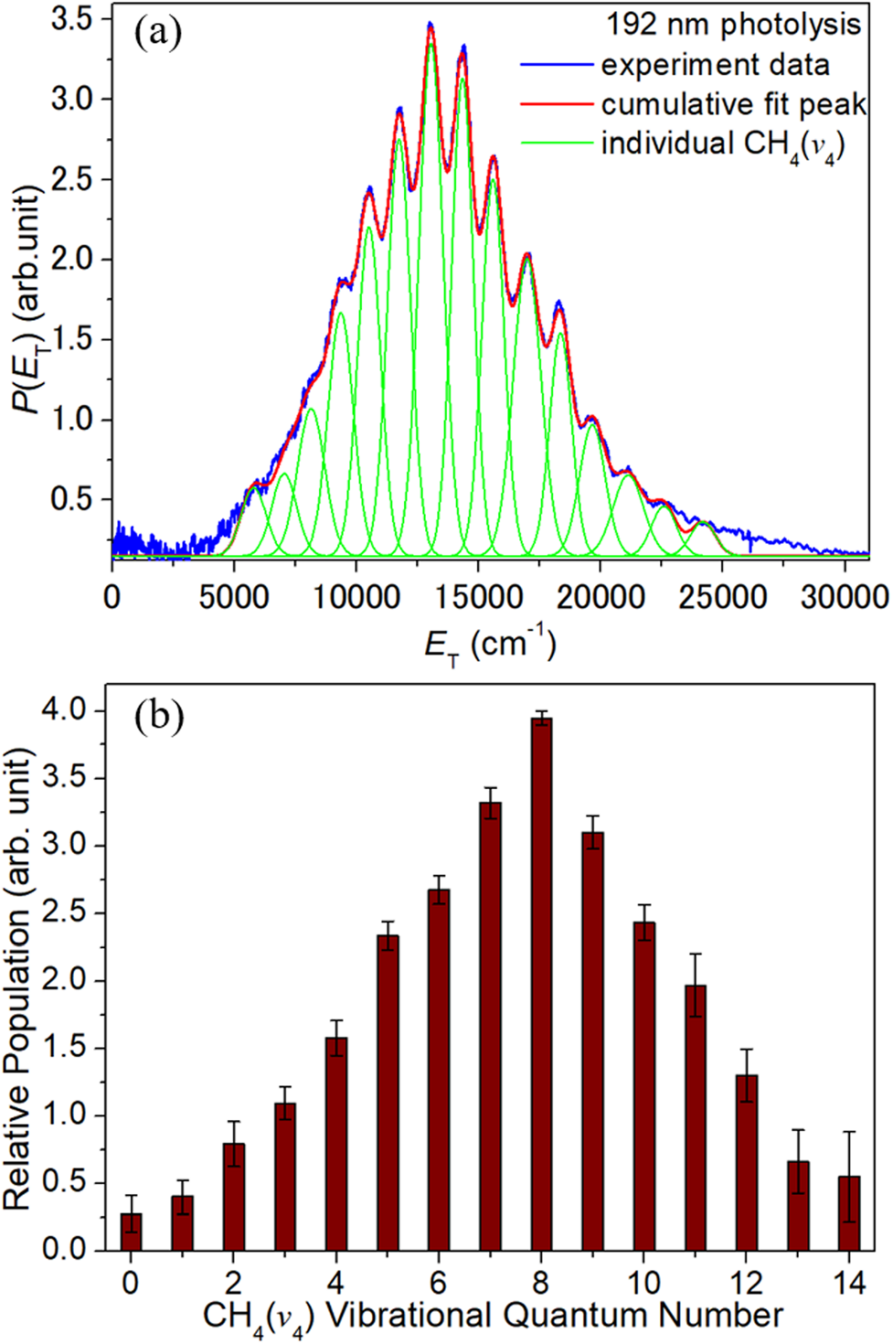
(a) Illustrative decomposition of the *P*(*E*_T_) spectrum obtained from analysis of the TS-VM image of the S(^1^D) fragments formed by photolysis of jet-cooled CH_3_SH molecules at *λ* = 192 nm using Gaussian functions to describe the relative populations in the partner CH_4_(*v*_4_) levels. (b) Histogram illustrating the inverted CH_4_*P*(*v*_4_) vibrational population distribution so derived. The error bars represent the standard error in the simulation.

The observed peaks align fairly well with the *E*_T(max)_ values predicted given the respective UV photon energies, *E*_th_(4) and the predicted wavenumber of the centre of gravity of each polyad with *n* ≤ 9,^65^ but the alignment between the ticks and the peaks degrades with increasing *n*. Better alignment is achieved by assuming a smaller peak spacing of ∼1330 cm^−1^, shown by the combs superposed above the data in Fig. 4(c) and (d) and in the right hand panels in Fig. 5. This hints that the observed peaks are dominated by a sub-set of levels involving multiples of the *ν*_4_ bending mode. Such an interpretation also fits better with the apparent near-constancy of the peak widths, the narrowness of which also implies that the CH_4_ fragments must be formed with only modest rotational excitation. As Fig. 6 shows, the vibrational state population distribution, *P*(*v*_4_), in the CH_4_ fragments formed when photolyzing at *λ* = 192 nm (Fig. 4(a)) is highly inverted, peaking at *v*_4_ = 8 and implying an average vibrational energy content in the CH_4_ fragments of ∼10 000 cm^−1^. The increased relative contribution to the S(^1^D) product yield from two-photolysis-photon induced dissociations in images recorded at longer wavelengths makes it hard to define any *λ*-dependence of the *P*(*v*_4_) distribution. The possible UV photofragmentation dynamics responsible for the deduced S(^1^D) + CH_4_ product state distribution is discussed along with the previously recognised dissociation processes (1) and (2) in the following section.

## 4. Discussion

The observation and characterization of the hitherto unreported S(^1^D) + CH_4_ product channel (4) following photoexcitation of CH_3_SH in the wavelength range 191 ≤ *λ* ≤ 204 nm are the most striking findings from the present work. In what follows, we consider possible mechanisms for this process – effectively an elimination of the central atom in an ABC framework to yield B + AC products. Reports of such photo-induced eliminations are becoming increasing commonplace,^52^ having recently been recognised in, for example: H_2_O (yielding H_2_ + O(^1^S) products),^67^ H_2_S (yielding H_2_ molecules together with a sulfur atom, in the ^1^D or ^1^S excited state),^56,68^ CO_2_,^69^ OCS,^70^ CS_2_,^51^ (yielding C(^3^P) atoms in each case), SO_2_ (yielding S(^1^D) products)^71^ and, in a molecule of more similar size to that of present interest, CH_3_NH_2_ (yielding NH(X^3^Σ^−^) + CH_4_ products).^72,73^ These prior studies suggest various (molecule specific) mechanisms for such photo-induced eliminations, amongst which are a class of frustrated dissociations sometimes described as ‘roaming’.^74^ In such cases, the topography of the excited state potential accessed by photoexcitation drives an initial bond extension, but the available energy is insufficient to enable complete bond fission. The partner moieties are thus drawn back towards one another and undergo what is effectively an intramolecular collision prior to re-separating as (potentially) an alternative product pair. The available data suggest that such a mechanism might account for the S(^1^D) + CH_4_ fragment channel identified in the present work.

First, we reiterate that the operation of rival primary C–S and S–H bond fission processes following photoexcitation to the 2^1^A′′ state at *λ* ∼200 nm is well-established. The adiabatic potential of the 2^1^A′′ state is bound in both *R*_C–S_ and *R*_S–H_; dissociation occurs on the 1^1^A′′ PES, after non-adiabatic coupling in an extended region of CI between the 2^1^A′′ and 1^1^A′′ potentials.^38,39^ The present work reveals some vibrational excitation in the SH(X) fragments formed *via* channel (1) when excited in the 190 ≤ *λ* ≤ 200 nm range, but also suggests that – as at longer excitation wavelengths – most of the photon energy in excess of that required to break the C–S bond is partitioned into CH_3_(X̃) + SH(X) product translational motion.^35,40–42^ The CH_3_S(X̃) products arising *via* the rival channel (2) following excitation to the 2^1^A′′ state and subsequent non-adiabatic coupling to the 1^1^A′′ PES are distributed over many vibrational levels, with very obvious activity in the *ν*_3_(C–S) stretch mode, but, again, even the most internally excited H + CH_3_S(X̃) products recoil with *E*_T_ ∼1 eV.^33,34^ Neither of these pathways obviously satisfy the picture of a frustrated dissociation and re-collision.

Several other points merit note, however. The relative importance of the C–S bond fission process (1) increases markedly once exciting in the 2^1^A′′ ← X̃^1^A′ absorption band, suggesting that a larger fraction of the molecules photoexcited at these shorter wavelengths undergo initial C–S bond extension. This accords with the observed *ν*_5_(C–S) stretch progression in the weak emission spectrum from the parent CH_3_SH molecules following excitation at *λ* = 193.3 nm.^36^ Additionally, the 2^1^A′′ PES is adiabatically bound, correlating to CH_3_(X̃) + SH(A) fragments when extending *R*_C–S_ (and to H + CH_3_S(Ã) fragments when extending *R*_S–H_). Thus, it is tempting to speculate that the observed S(^1^D) + CH_4_ products might arise from a fraction of the photoexcited molecules that initially distort by extending *R*_C–S_, avoid non-adiabatic coupling to the 1^1^A′′ PES during this bond extension phase, reach the maximum *R*_C–S_ value consistent with energy conservation on the 2^1^A′′ PES, then re-compress to enable a re-encounter between the CH_3_ and SH moieties and, potentially, an exothermic reaction on to S(^1^D) + CH_4_ products (4).

Further mechanistic speculation at this stage is unwarranted, but it is very much hoped that the present data will inspire new high-level quantum chemical calculations of the 2^1^A′′ and 1^1^A′′ PESs and the non-adiabatic coupling between them (and, conceivably, to the X̃^1^A′ PES), together with *ab initio* molecular dynamics calculations designed to explore further the dynamics displayed by the products of dissociation processes (1), (2), (4) and, potentially, (3) and (5) – all of which could plausibly arise when exciting CH_3_SH at *λ* ∼200 nm. Future studies (experimental and/or theoretical) might also usefully search for processes yielding S(^3^P) atom products, estimate product quantum yields (branching fractions), and explore how these vary with the excitation wavelength. Extension of such contemporary photofragmentation studies to the oxygen analogue, methanol, the simplest and most abundant interstellar complex organic molecule observed in warm and cold environments,^75,76^ should be an obvious priority, but searching for singlet products (*e.g*. NH(a^1^Δ) + CH_4_) from photolysis of methylamine could also be very rewarding.

## Author contributions

K. J. Y. and X. M. Y. supervised the research. K. J. Y. conceived the research. M. N. R. A. and K. J. Y. designed the experiments. Y. C. W., S. Y. Z., Z. J. L., S. K. Y., Z. X. L., Y. X. D., W. H. and Q. S. performed the experiments. Y. C. W., D. X. D., M. N. R. A., K. J. Y and X. M. Y. analysed the data. Y. C. W., K. J. Y. and M. N. R. A. wrote the manuscript. All authors discussed the results and commented on the manuscript.

## Conflicts of interest

There are no conflicts to declare.

## Supplementary Material

SC-OLF-D5SC04716A-s001

## Data Availability

The data supporting this study are available within the main text and the SI. See DOI: https://doi.org/10.1039/d5sc04716a.
