# Supplementary material for: Ultraviolet photodissociation of methanethiol (CH3SH): revealing an S(1D) atom elimination channel
Source: Chem Sci. 2025 Aug 18;16(37):17165–75. doi: 10.1039/d5sc04716a (PMC12396137; doi:10.1039/d5sc04716a)
Supplement: SC-016-D5SC04716A-s001 [file SC-016-D5SC04716A-s001.pdf]

Supplementary Information (SI) for Chemical Science.
This journal is © The Royal Society of Chemistry 2025

Electronic Supplementary Information for:

Ultraviolet photodissociation of methanethiol (CH_3SH): Revealing an S(^{1}D) atom elimination channel

Yucheng Wu[1,2], Shunyang Zhou[1], Zijie Luo[1,3], Shuaikang Yang[1], Zhenxing Li[4], Yongxin Dong[3], Wei Hua[1], Quan Shuai[1], Dongxu Dai[1], Michael N.R. Ashfold[5*], Kaijun Yuan[1,2,6*], Xueming Yang[1, 6,7]

1. State Key Laboratory of Chemical Reaction Dynamics and Dalian Coherent Light Source, Dalian Institute of Chemical Physics, Chinese Academy of Sciences, 457 Zhongshan Road, Dalian, 116023, China.
2. University of Chinese Academy of Sciences, Beijing 100049, China.
3. Marine Engineering College, Dalian Maritime University, Liaoning, 116026, China.
4. Institute of Advanced Light Source Facilities, Shenzhen, Guangdong, 518100, China.
5. School of Chemistry, University of Bristol, Bristol, BS8 1TS, U.K.
6. Hefei National Laboratory, Hefei 230088, China.
7. Department of Chemistry and Center for Advanced Light Source Research, College of Science, Southern University of Science and Technology, Shenzhen 518055, China.

* To whom correspondence should be addressed. E-mail addresses: kjyuan@dicp.ac.cn. mike.ashfold@bristol.ac.uk

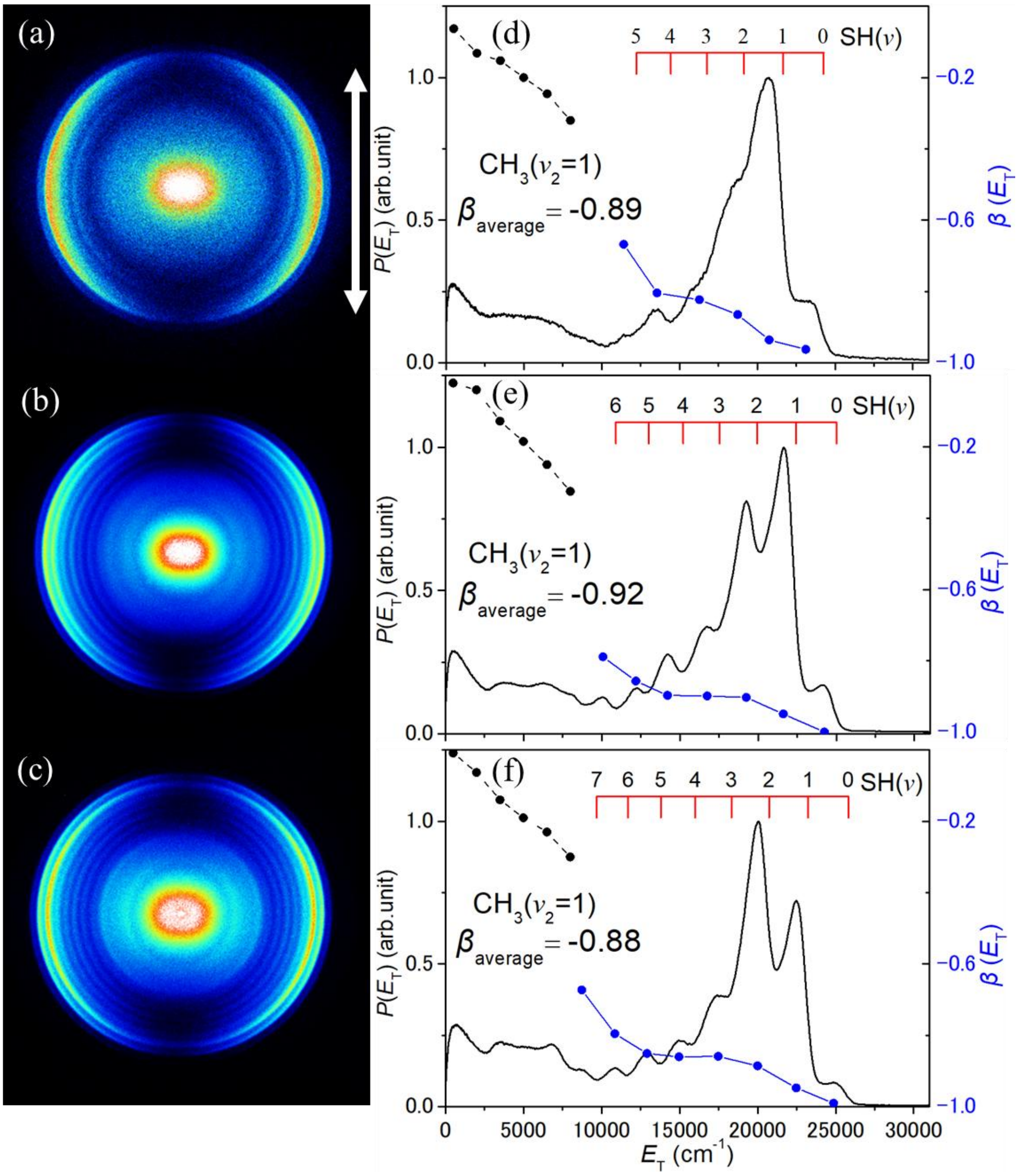

Figure S1. TS-VM images of $CH_3(v_2=1)$ fragments formed by photolysis of jet-cooled CH_3SH molecules at λ = (a) 199, (b) 196 and (c) 193 nm, with $\boldsymbol{\varepsilon}_{\mathbf{phot}}$ aligned vertically in the plane of the image as indicated by the double headed arrow in (a). The corresponding $P(E_T)$ (black) and $\beta(E_T)$ (blue) distributions derived from each image are displayed in panels (d), (e) and (f), with the relevant scales shown on, respectively, the left- and right-hand y-axes, along with the $\beta_{average}$ value determined over the indicated E_T range. The red combs above the respective $P(E_T)$ spectra indicate the maximum E_T value for the specified co-fragment formed with the probed $CH_3(v_2=1)$ species.

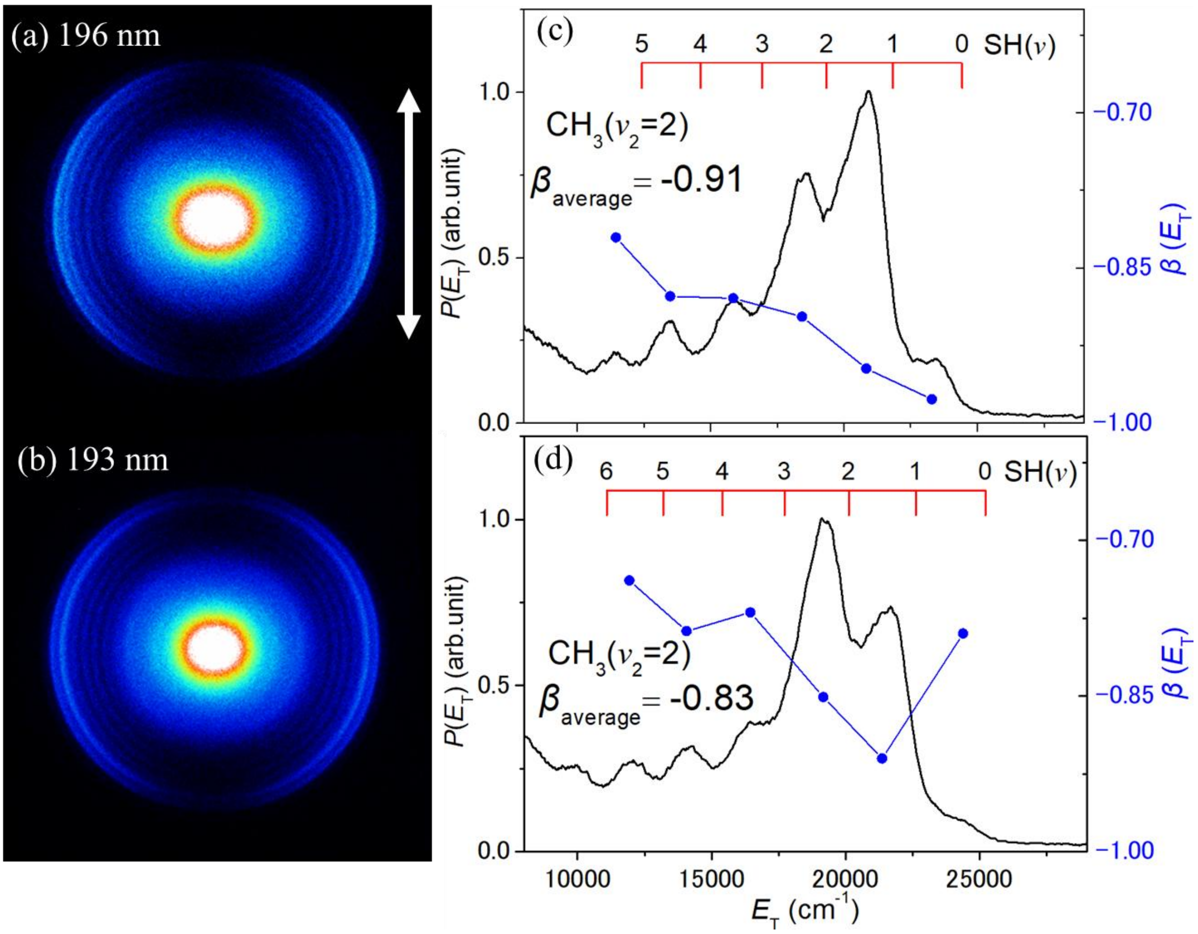

Figure S2. TS-VM images of $CH_3(v_2=2)$ fragments formed by photolysis of jet-cooled CH_3SH molecules at λ = (a) 196 and (b) 193 nm, with $\boldsymbol{\varepsilon}_{\mathbf{phot}}$ aligned vertically in the plane of the image as indicated by the double headed arrow in (a). The corresponding $P(E_T)$ (black) and $\beta(E_T)$ (blue) distributions derived from these images are displayed in panels (c) and (d), with the relevant scales shown on, respectively, the left- and right-hand y-axes, along with the $\beta_{average}$ value determined over the indicated E_T range. The red combs above the respective $P(E_T)$ spectra indicate the maximum E_T value for the specified co-fragment formed with the probed species.

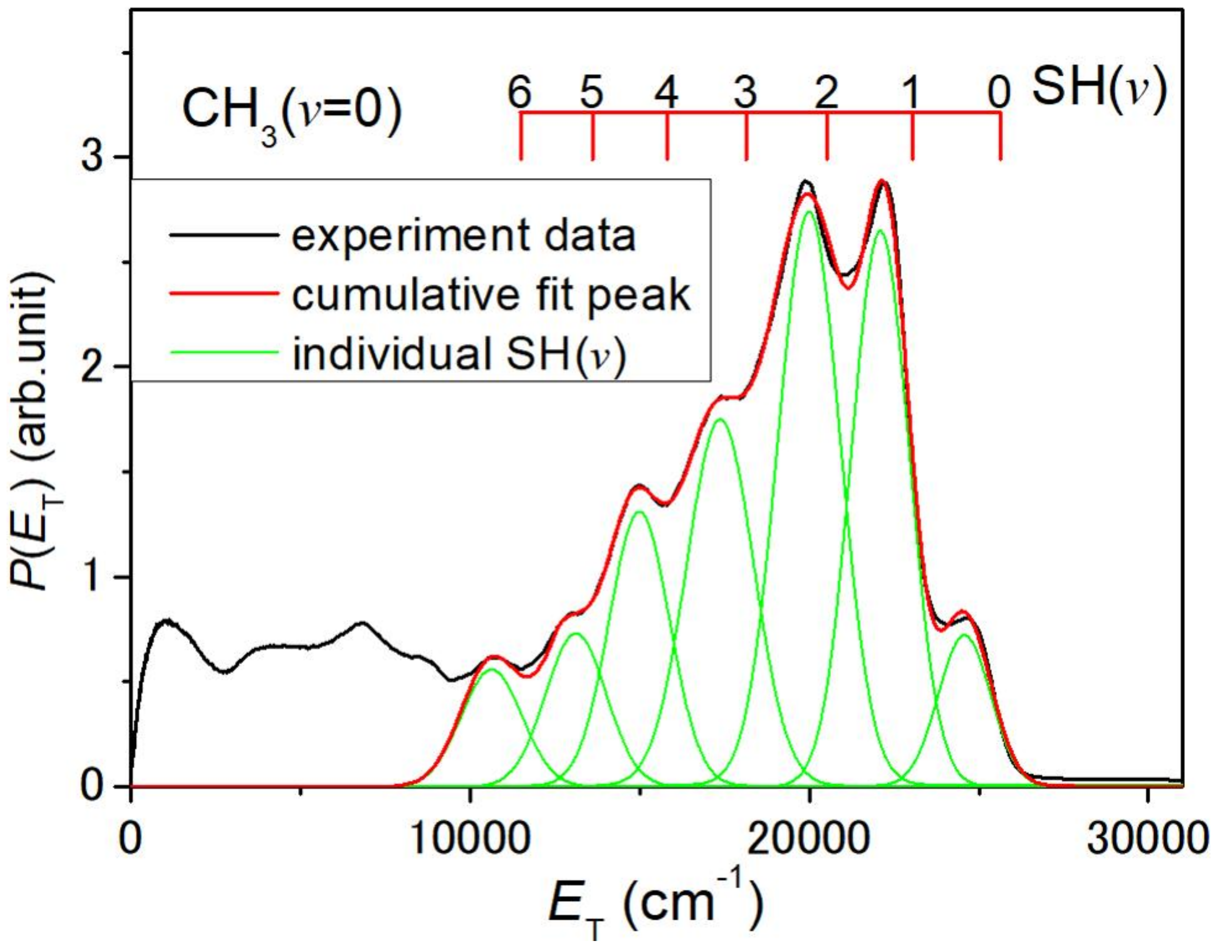

Figure S3. Illustrative decomposition of the $P(E_T)$ spectrum obtained from analysis of the TS-VM image of the CH_3(v=0) fragments formed by photolysis of jet-cooled CH_3SH molecules at λ = 196 nm using Gaussian functions to describe the relative populations in the partner SH(v) levels. The red comb indicates the maximum E_T value for the specified level SH(v) formed with the probed species. The signal at low E_T, attributed to secondary photolysis of CH_3S(X) fragments arising via rival dissociation channel (2) surely extends to E_T values that overlap with the primary CH_3 + SH(X, higher v) fragments, implying that the decomposition displayed here will overestimate the relative yield of SH(X, v = 6 (and 5)) fragments.

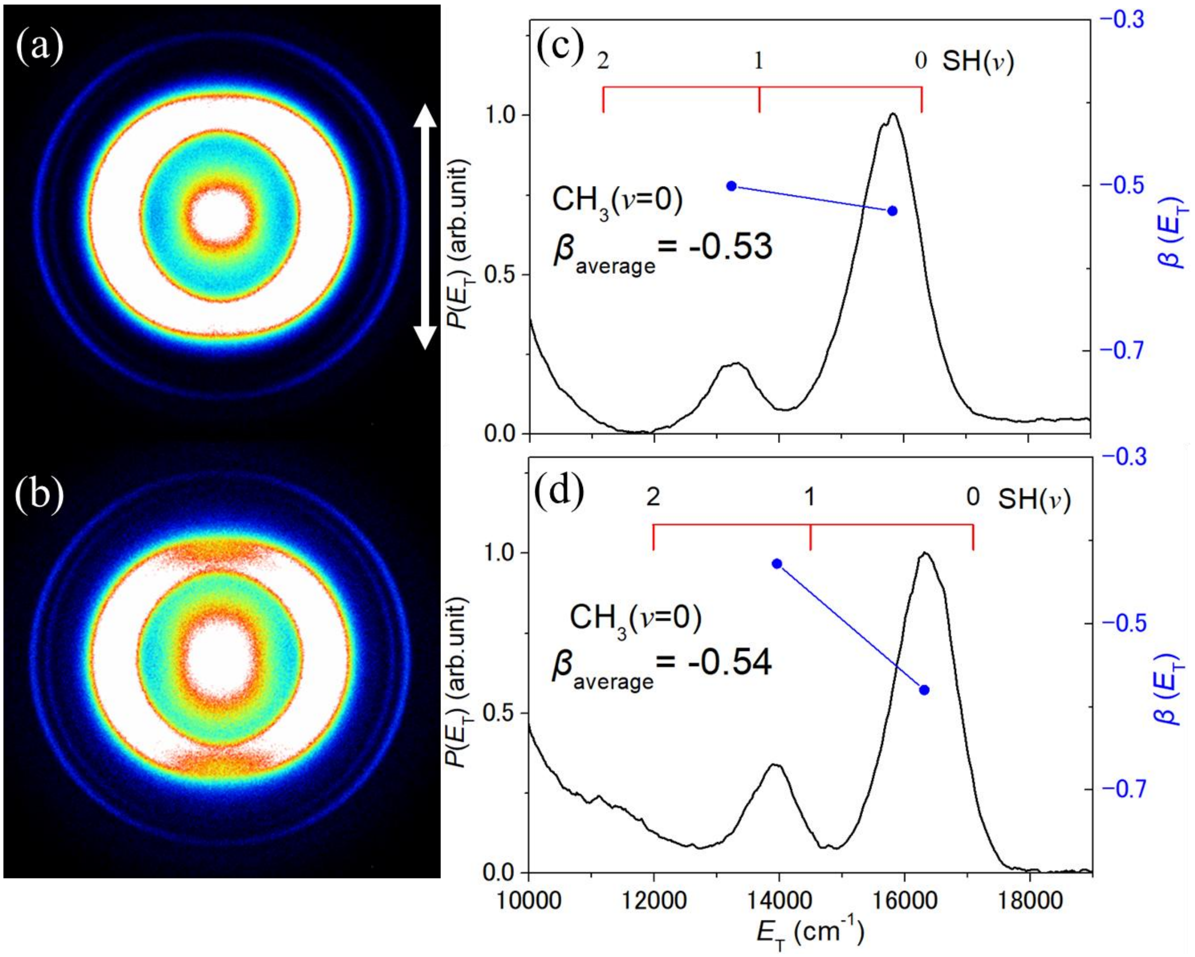

Figure S4. TS-VM images of CH_3(v=0) fragments formed by photolysis of jet-cooled CH_3SH molecules at λ = (a) 240 and (b) 235 nm, with $\boldsymbol{\varepsilon}_{\mathbf{phot}}$ aligned vertically in the plane of the image as indicated by the double headed arrow in (a). The partially saturated signal in the middle of the image is attributed to CH_3 fragments from secondary photolysis of CH_3S(X) fragments arising via rival dissociation channel (2). The higher-E_T part of the corresponding $P(E_T)$ (black) and $\beta(E_T)$ (blue) distributions derived from these images are displayed in panels (c) and (d), with the relevant scales shown on, respectively, the left- and right-hand y-axes, along with the $\beta_{average}$ value determined over the indicated E_T range. The red combs above the respective $P(E_T)$ spectra indicate the maximum E_T value for the specified co-fragment formed with the probed species.

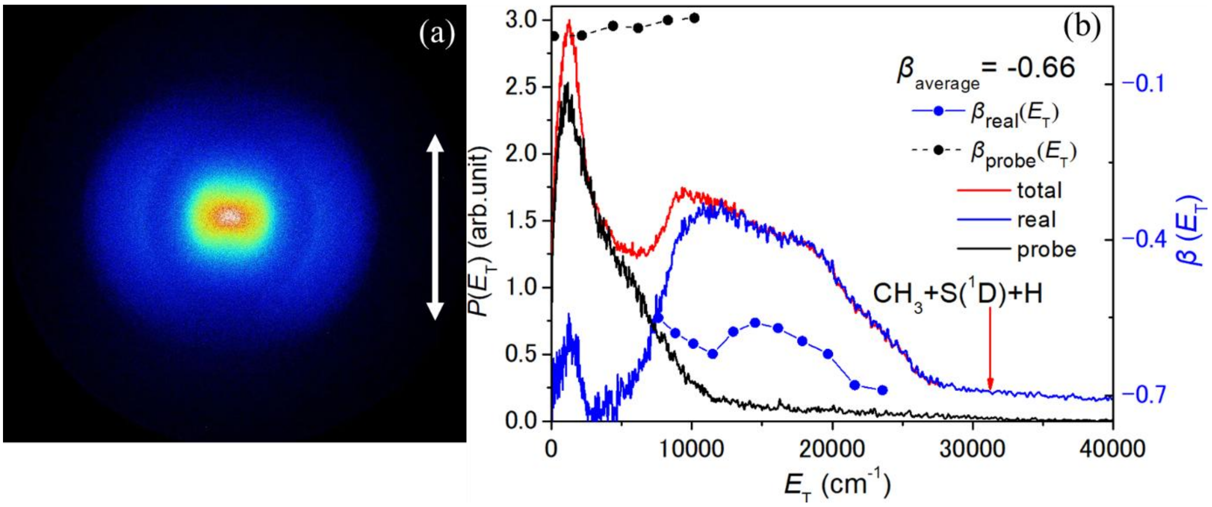

Figure S5. (a) TS-VM image of the S(^{1}D) fragments formed by photolysis of jet-cooled CH_3SH molecules at λ= 212 nm, with $\boldsymbol{\varepsilon}_{\mathbf{phot}}$ aligned vertically in the plane of the image as indicated by the double headed arrow. (b) $P(E_T)$ distributions derived from this two-color image (red trace), from the 130.091 nm probe-laser only image (black trace) and the 'real' pump-probe two-color $P(E_T)$ distribution obtained from the difference (blue trace), referenced to the left-hand *y*-axis scale. When exciting at λ= 212 nm, the latter signal is wholly attributed to two-photolysis-photon induced dissociation of CH_3SH (process (10)). The black and blue dots show the $\beta(E_T)$ distributions derived from the corresponding traces, referenced to the right-hand *y*-axis scale. The $\beta_{average}$ value for the signal from two-photolysis-photon induced dissociation determined over the indicated E_T range is also included in the inset.
